# Inflammatory score as a predictor of survival and nutritional deterioration in cancer patients: insights from a multicenter cohort study

**DOI:** 10.3389/fnut.2025.1631483

**Published:** 2025-08-04

**Authors:** Hailun Xie, Shuyao Wang, Lishuang Wei, Siyu Lin, Hanping Shi, Junqiang Chen

**Affiliations:** ^1^Department of Gastrointestinal and Gland Surgery, The First Affiliated Hospital, Guangxi Medical University, Nanning, Guangxi, China; ^2^Guangxi key Laboratory of Enhanced Recovery After Surgery for Gastrointestinal Cancer, The First Affiliated Hospital of Guangxi Medical University, Nanning, Guangxi, China; ^3^Guangxi Clinical Research Center for Enhanced Recovery After Surgery, The First Affiliated Hospital of Guangxi Medical University, Nanning, Guangxi, China; ^4^Guangxi Zhuang Autonomous Region Engineering Research Center for Artificial Intelligence Analysis of Multimodal Tumor Images, The First Affiliated Hospital of Guangxi Medical University, Nanning, Guangxi, China

**Keywords:** inflammatory score, overall survival, malnutrition, cachexia, cancer

## Abstract

**Background and aims:**

Chronic inflammation is a hallmark of cancer progression. This multicenter cohort study aimed to evaluate the prognostic value of a novel inflammatory score, derived from baseline white blood cell (WBC) count and C-reactive protein (CRP) z-scores, in predicting survival outcomes and nutritional deterioration among cancer patients.

**Methods:**

We analyzed data from 6,568 cancer patients across multiple institutions. The inflammatory score was categorized as mild, moderate, or severe. Kaplan–Meier survival analysis, Cox proportional hazards models, and restricted cubic splines were used to assess associations with all-cause mortality. Subgroup analyses were stratified by tumor type and pathological stage. Logistic regression models quantified associations between inflammatory scores and nutritional deterioration.

**Results:**

Dose–response analyses revealed a nonlinear relationship between continuous inflammatory scores and mortality (HR = 1.200, 95% CI: 1.163–1.238, *p* < 0.001). Higher inflammatory scores were significantly associated with reduced survival (67.5% vs. 65.3% vs. 57.0% vs. 45.2%, *p* < 0.001). In fully adjusted models, severe inflammation conferred a 60.4% increased mortality risk (HR = 1.604, 95% CI: 1.464–1.757, *p* < 0.001) compared to mild inflammation. Subgroup analyses confirmed consistent associations across tumor types and pathological stages. Advanced-stage (III/IV) patients exhibited heightened sensitivity to inflammatory burden, underscoring its role in late-stage prognosis. Severe inflammation was also linked to higher rates of severe malnutrition (OR = 2.553, 95%CI: 2.226–2.927, *p* < 0.001) and cachexia (OR = 2.662, 95%CI: 2.323–3.049, *p* < 0.001). Validation cohorts reproduced these findings, underscoring the score’s robustness.

**Conclusion:**

The inflammatory score, integrating WBC and CRP, is a strong independent predictor of survival and nutritional deterioration in cancer patients. Its clinical utility for risk stratification and guiding targeted anti-inflammatory therapies warrants further exploration.

## Introduction

Cancer remains a leading cause of global morbidity and mortality, imposing profound physical, emotional, and socioeconomic burdens on patients, families, and healthcare systems (1, 2). Emerging evidence underscores the pivotal role of chronic inflammation in driving cancer initiation, progression, and metastasis (3–5). A pro-inflammatory tumor microenvironment, characterized by cytokine-driven immune dysregulation, angiogenesis, and tissue remodeling, not only fuels tumor growth but also facilitates immune evasion and distant spread, significantly impairing patient prognosis (6, 7). While traditional prognostic tools such as tumor stage and histology provide foundational insights, they often fail to capture the systemic biological complexity of cancer, particularly the dynamic interplay between inflammation and disease progression.

Systemic inflammatory biomarkers, including white blood cell (WBC) count and C-reactive protein (CRP), have emerged as critical indicators of tumor-associated inflammation. Elevated levels of these markers reflect a hostile microenvironment enriched with pro-tumor cytokines (e.g., IL-6, TNF-*α*) and correlate with poor survival across diverse malignancies, such as lung, gastrointestinal, and breast cancers (8–10). These biomarkers have been independently associated with poor survival across multiple malignancies, including lung, gastrointestinal, and breast cancers. However, individual inflammatory markers often fall short in fully and accurately capturing the complex inflammatory status within patients. There remains a need for a reliable and stable composite inflammatory index to reflect the overall inflammatory state in cancer patients. Composite scores, such as the neutrophil-to-lymphocyte ratio (NLR) and platelet-to-lymphocyte ratio (PLR), have shown promise in improving prognostic accuracy but lack standardization and fail to integrate key inflammatory mediators (11–13). To address these gaps, recent advancements propose novel inflammatory scores that combine multiple biomarkers, such as baseline WBC and CRP levels standardized as z-scores, to holistically quantify inflammatory burden (14, 15). These scores offer enhanced stability and clinical utility, enabling more precise risk stratification and monitoring of therapeutic responses.

Compounding the prognostic challenge is the bidirectional relationship between chronic inflammation and cancer-related malnutrition. Pro-inflammatory cytokines, including IL-6 and TNF-*α*, drive muscle catabolism and anorexia, contributing to cachexia—a debilitating condition affecting 30–50% of cancer patients and strongly associated with mortality (16, 17). Despite this, few large-scale studies have comprehensively evaluated how systemic inflammation interacts with nutritional deterioration to influence survival, particularly across heterogeneous tumor types and pathological stages.

Given the current landscape, the present study aims to leverage a multicenter cohort of 6,568 cancer patients to thoroughly investigate the prognostic value of the inflammatory score for all-cause mortality across diverse malignancies and pathological stages. Additionally, we also investigate the association between inflammatory burden and nutritional outcomes, including severe malnutrition and cachexia. This study provides robust evidence on the interplay between systemic inflammation, survival, and cancer nutritional status, offering potential applications for guiding clinical treatment decisions and optimizing patient management strategies. It aims to provide a more targeted and effective basis for clinical practice in cancer care.

## Materials and methods

### Study design and population

This multicenter retrospective study, consistent with prior research protocols, enrolled cancer patients from multiple participating institutions. The names of all participating hospitals were shown in Supplementary Table S1. The study protocol received ethical approval from the Institutional Review Board of the First Affiliated Hospital of Guangxi Medical University (2025-E0566). Inclusion criteria comprised: (1) histologically or cytologically confirmed malignancies (including lung, gastrointestinal, breast, and other solid tumors); (2) age ≥ 18 years; (3) availability of complete clinicopathological data; and (4) provision of written informed consent. Exclusion criteria included: (1) severe comorbidities (e.g., advanced cardiovascular disease, hepatic or renal failure) that might confound survival outcomes; (2) psychiatric disorders impairing compliance with study assessments; or (3) recent immunotherapies or other treatments potentially altering inflammatory status. The study strictly adhered to ethical principles outlined in the *Declaration of Helsinki*. All eligible patients and their legal guardians provided written informed consent after receiving detailed explanations of the study.

### Data collection

Baseline demographic, clinical, and laboratory data were prospectively collected, encompassing age, sex, TNM stage (AJCC 8th edition), tumor type (Lung and bronchus, Esophagus, Gastric, Liver and intrahepatic bile duct, Pancreatic, Colon & rectum, Breast, Gynecological, Urologic, Nasopharynx, and Brain), treatment modalities (surgery, radiotherapy, chemotherapy), comorbidities (hypertension, diabetes), lifestyle factors (smoking, alcohol use), family cancer history, Karnofsky Performance Status (KPS) and nutritional assessments (Patient-Generated Subjective Global Assessment [PG-SGA] score, Nutritional Risk Screening 2002 [NRS2002], and cachexia diagnosis). Venous blood samples were obtained at enrollment and analyzed using standardized assays to quantify white blood cell (WBC) count, neutrophil count, lymphocyte count, platelet count, red blood cell (RBC) count, hemoglobin levels, as well as C-reactive protein (CRP) and albumin concentrations.

### Inflammatory score calculation

Based on previous research, the inflammatory score was computed as the sum of standardized z-scores for baseline WBC count and CRP levels (14, 15). The population mean (*μ*) and population standard deviation (*σ*) for WBC (μ = 6.64 × 10^9^/L, σ = 3.06) and CRP (μ = 19.41 mg/L, σ = 40.77) were derived from the distribution of all enrolled patients at baseline. For each biomarker, z-scores were calculated using the formula:


$$z-\text{score}=\frac{\text{Individual value}-\text{Population mean}}{\text{Population standard deviation}}$$

In prior studies, total scores were categorized into quartiles: *Q1* (lowest quartile), *Q2* (second quartile), *Q3* (third quartile), and *Q3* (highest quartile). For this study, the *Q1* and *Q2* categories were consolidated into a single *mild* group, resulting in three classifications: *mild*, *moderate*, and *severe* inflammation.

### Survival data collection and study outcomes

Patients underwent structured follow-up assessments: every 3 months for the first 2 years post-treatment to monitor potential recurrence or metastasis, every 6 months from years 3 to 5 to track disease progression, and annually thereafter for long-term survival surveillance. Follow-up strategies included in-person clinic visits supplemented by telephone interviews and digital platforms. The primary outcome was overall survival (OS), defined as the duration from cancer diagnosis to death from any cause, loss to follow-up, or study termination, whichever occurred first. This endpoint comprehensively reflects the entire survival trajectory from diagnosis to mortality, serving as a critical metric for evaluating therapeutic efficacy and prognosis. Secondary outcomes included severe malnutrition (defined as PG-SGA score > 8) and cachexia, diagnosed according to the 2023 Asian Working Group for Cachexia (AWGC) Consensus criteria (18–20).

### Statistical analysis

Continuous variables with normal distributions are presented as mean ± standard deviation (Mean ± SD), and between-group comparisons were performed using independent *t*-tests, following verification of normality via Shapiro–Wilk tests (*p* > 0.05). Non-normally distributed continuous variables are expressed as median (interquartile range) [M (IQR)], with between-group differences assessed via nonparametric Mann–Whitney U tests (two groups) or Kruskal-Wallis H tests (multiple groups), supplemented by Dunn’s *post hoc* tests for pairwise comparisons. Categorical variables are reported as frequencies (percentages) [*n* (%)], and between-group differences were evaluated using chi-square (χ^2^) tests or Fisher’s exact tests when expected cell counts were <5. Ordinal data were analyzed using nonparametric rank-sum tests to adjust for confounding factors. Kaplan–Meier curves and log-rank tests compared survival differences across inflammatory categories. Cox proportional hazards regression models were employed to calculate hazard ratios (HRs) and 95% confidence intervals (CIs) for associations between inflammatory scores and overall survival (OS). Three hierarchical models were constructed: *Model a* (unadjusted), *Model b* (adjusted for age, sex, BMI, TNM stage, and tumor types), and *Model c* (further adjusted for surgery, radiotherapy, chemotherapy, hypertension, diabetes, smoking, drinking, and family history). Covariates for adjusted models were selected based on: (i) Established prognostic factors in oncology (age, sex, BMI, TNM stage, tumor types); (ii) Treatments directly modulating inflammation or survival (surgery, radiotherapy, chemotherapy); (iii) Comorbidities/lifestyle factors confounding inflammation-nutrition interactions (hypertension, diabetes, smoking, drinking, family history). These variables were prioritized due to clinical relevance and data availability across all centers. Nonlinear associations between continuous inflammatory scores and mortality were assessed using restricted cubic splines (RCS) with four knots. Stratified Cox models evaluated interactions by tumor type. To address immortal time bias, patients who died within 90 days (*n* = 419) were excluded in sensitivity analyses. Logistic regression models quantified associations between inflammatory scores and severe malnutrition (PG-SGA > 8) or cachexia, generating odds ratios (ORs) with 95% CIs. Internal validation involved splitting the cohort into derivation and validation subsets (7:3 ratio) to test result robustness. Statistical significance was defined as two-sided *p* < 0.05. All analyses were conducted using R statistical software (Version 4.3.1; R Foundation for Statistical Computing), and graphical visualizations were generated using GraphPad Prism 10.1.2 (GraphPad Software).

## Results

### Baseline characteristics and inflammatory score distribution

The study cohort comprised 6,568 cancer patients (60.5% male; mean age 59.5 ± 11.2 years) with diverse malignancies, including lung and bronchus (32.2%), colorectal (19.7%), and gastric cancers (15.2%). Baseline characteristics stratified by inflammatory score categories (mild, moderate, severe) are detailed in Supplementary Table S2. Patients with severe inflammatory scores exhibited a higher proportion of males, advanced age, and elevated rates of hypertension, diabetes, smoking, and alcohol use (all *p* < 0.05). Advanced TNM staging was more prevalent in the severe group (Stage IV: 55.7% vs. 38.8% in mild group, *p* < 0.001), suggesting a strong association between inflammatory burden and disease progression. Tumor type distribution varied significantly: lung, pancreatic, and brain/central nervous system tumors predominated in the severe group, whereas breast and nasopharyngeal cancers were more common in the mild group (all *p* < 0.001). Treatment modalities also differed, with higher rates of surgery (67.8% vs. 49.9%), radiotherapy (16.4% vs. 10.5%), and chemotherapy (73.4% vs. 58.8%) in the mild group compared to the severe group (all *p* < 0.001). Laboratory findings revealed elevated white blood cells, neutrophils, and platelets, alongside reduced lymphocytes, hemoglobin, and albumin levels in the severe group (all *p* < 0.001), indicative of heightened systemic inflammation and compromised nutritional status. Furthermore, severe inflammation was associated with higher rates of cachexia (52.8% vs. 27.2%), 90-day adverse outcomes (13.0% vs. 3.1%), mortality (54.8% vs. 33.6%), and hospitalization costs (median ¥21,698 vs. ¥15,306), alongside poorer PG-SGA and KPS scores (all *p* < 0.001), underscoring the clinical and economic impact of inflammatory burden.

### Kaplan–Meier survival analysis by inflammatory score

**Figure 1:**
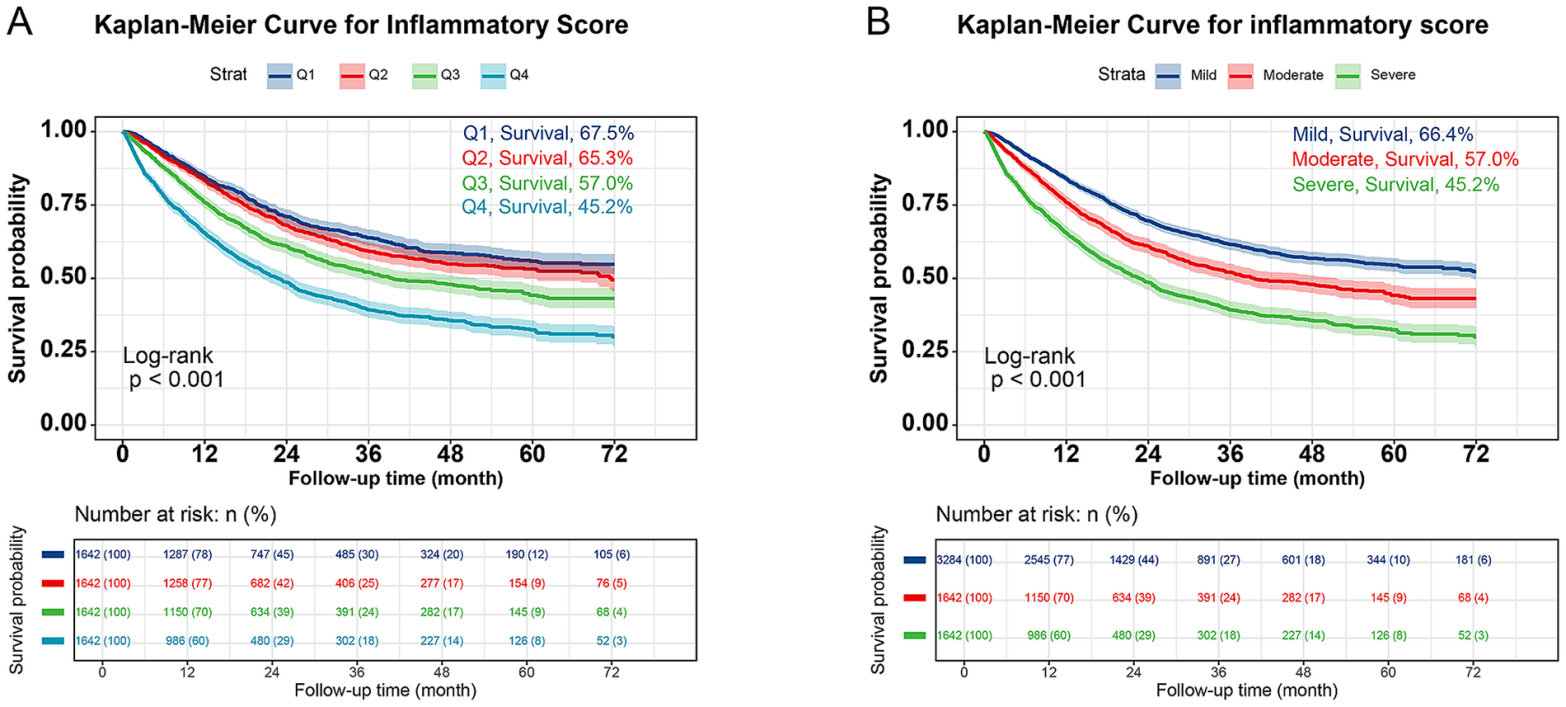
Kaplan–Meier curve of cancer patients with different levels of inflammatory score. **(A)** Quantiles of the inflammatory score; **(B)** Three classifications of inflammatory score.

Kaplan–Meier survival curves demonstrated significant survival disparities across inflammatory score categories (67.5% vs. 65.3% vs. 57.0% vs. 45.2%, *log-rank p* < 0.001; Figure 1A). Given minimal survival differences between the original *Q1* and *Q2* groups (67.5% vs. 65.3%), these categories were merged into a single *mild* group. Subsequent analysis revealed a stepwise decline in survival with increasing inflammatory severity: 66.4% (mild), 57.0% (moderate), and 45.2% (severe) survival rates (*p* < 0.001; Figure 1B). Subgroup analyses stratified by TNM stage (Supplementary Figures S1A–D) highlighted stronger associations between inflammation and mortality in advanced stages. For Stage I patients, survival rates were 89.4% (mild), 87.8% (moderate), and 80.5% (severe) (*p* = 0.021); no significant difference was observed in Stage II (*p* = 0.063). In Stage III, survival declined sharply to 71.4% (mild), 63.2% (moderate), and 49.5% (severe) (*p* < 0.001), while Stage IV exhibited the most pronounced disparity (47.9% vs. 31.3%, *p* < 0.001). Early separation of survival curves in Stages III/IV within the severe group underscores the prognostic utility of inflammatory scoring for late-stage risk stratification.

### Association of inflammatory score with survival outcomes

**Figure 2:**
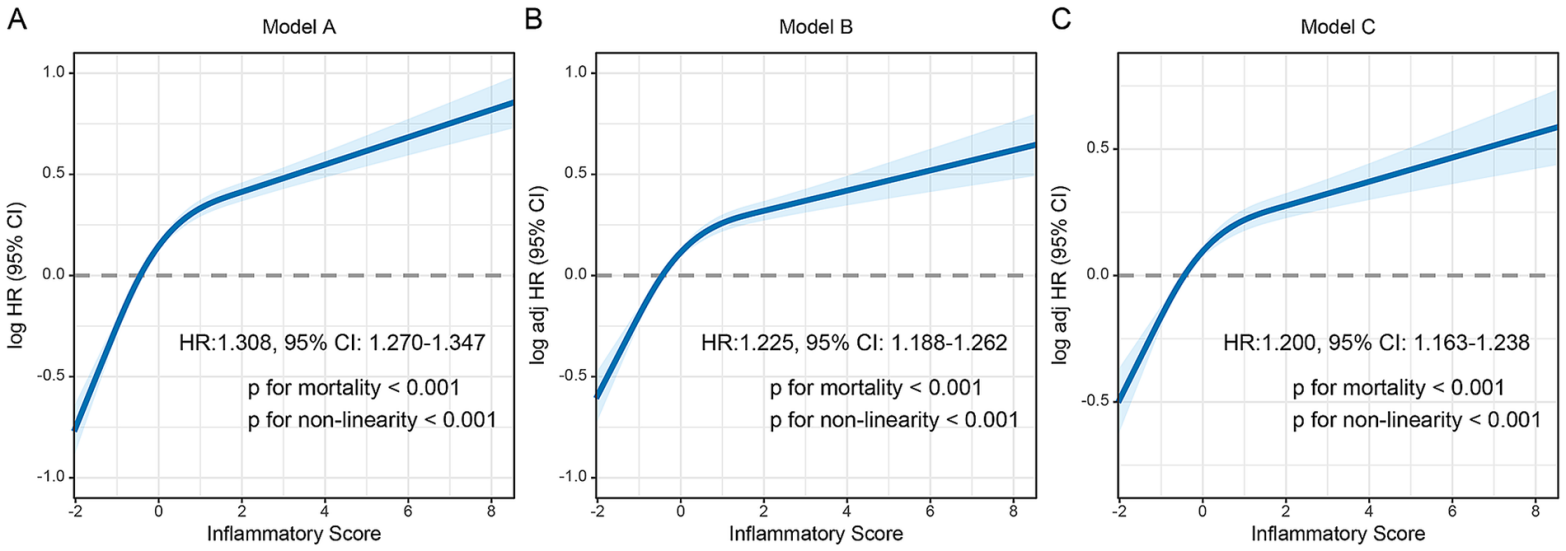
Dose–response relationship between inflammatory score and all-cause mortality in patients with cancer. **(A)** Model a: No adjusted. **(B)** Model b: Adjusted for age, sex, BMI, TNM stage, tumor types. **(C)** Model c: Adjusted for age, sex, BMI, TNM stage, tumor types, surgery, radiotherapy, chemotherapy, hypertension, diabetes, smoking, drinking, family history.

The Akaike Information Criterion (AIC) and Bayesian Information Criterion (BIC) values for the restricted cubic spline models indicate that the four knots model exhibits better goodness of fit to the data (Supplementary Table S3). RCS analysis confirmed a nonlinear dose–response relationship between continuous inflammatory scores and mortality (*P* for nonlinearity < 0.001; Figure 2). Mortality risk escalated modestly at lower scores but surged sharply in the severe range. Each SD increase in inflammatory score corresponded to a 16.3% higher mortality risk (HR = 1.200, 95% CI: 1.163–1.238, *p* < 0.001). The threshold value of the Inflammatory Score for predicting mortality was-0.440. When the score exceeded this threshold, the risk of all-cause mortality increased significantly with further elevations in the Inflammatory Score. Cox proportional hazards models revealed a dose-dependent mortality risk: In the Model a (Unadjusted), Severe inflammation conferred a 1.06-fold higher mortality risk (HR = 2.064, 95% CI: 1.890–2.254, *p* < 0.001). Adjusted for age, sex, and TNM stage, Risk attenuated but remained significant (HR = 1.727, 95% CI: 1.579–1.887, *p* < 0.001). Cox proportional hazards models revealed a graded mortality risk: unadjusted severe inflammation conferred a 1.064-fold risk (HR = 2.064, 95% CI: 1.890–2.254, *p* < 0.001), which attenuated but remained significant after adjusting for age, sex, and TNM stage (HR = 1.727, 95% CI: 1.579–1.887, *p* < 0.001). In the fully adjusted model (Model c), severe inflammation independently predicted a 60.4% increased mortality risk (HR = 1.604, 95% CI: 1.464–1.757, *p* < 0.001; Table 1). Sensitivity analysis demonstrated that the mortality risk associated with moderate and severe inflammatory scores remained significant after excluding patients who died within a short period (419 cases), indicating robustness of the results (Supplementary Table S4). The subgroup forest plot of tumor types revealed that, across nearly all cancer types, both moderate and severe inflammatory scores were associated with a higher risk of mortality, exhibiting a gradient relationship where higher inflammatory scores corresponded to increased mortality risk (Supplementary Figure S2). ROC curves showed that the Inflammatory Score exhibited superior prognostic predictive efficacy compared to NLR and PLR (3-year OS: 0.608 vs. 0.526 vs. 0.558; 5-year OS: 0.592 vs. 0.511 vs. 0.531) (Supplementary Figure S3).

**Table 1:** Association between inflammatory score and survival of patients with cancer.

Inflammatory score	Model a [HR, 95% CI]	*p* value	Model b [HR, 95% CI]	*p* value	Model c [HR, 95% CI]	*p* value
Mild	ref	<0.001	ref	<0.001	ref	<0.001
Moderate	1.391 (1.266,1.529)	<0.001	1.275 (1.16,1.402)	<0.001	1.212 (1.101,1.334)	<0.001
Severe	2.064 (1.890,2.254)	<0.001	1.727 (1.579,1.887)	<0.001	1.604 (1.464,1.757)	<0.001

Model a, No adjusted; Model b, Adjusted for age, sex, BMI, TNM stage, tumor types; Model c, Adjusted for age, sex, BMI, TNM stage, tumor types, surgery, radiotherapy, chemotherapy, hypertension, diabetes, smoking, drinking, family history.

### Inflammatory score distribution across tumor types

Significant variations in inflammatory scores were observed among tumor types (Supplementary Figure S4). Lung and bronchus cancers exhibited the highest median scores, reflecting intense systemic inflammation, whereas breast cancer patients had the lowest scores. This trend persisted across pathological stages (Supplementary Figure S5). Tumors were classified into three inflammatory burden categories: *high* (lung, pancreatic, brain), *moderate* (esophageal, gastric, hepatic, colorectal, urologic, gynecological), and *low* (breast, nasopharyngeal). Kaplan–Meier analysis revealed stark survival differences: 46.2% (severe), 62.3% (moderate), and 85.5% (low) survival rates (*log-rank p* < 0.001; Supplementary Figure S6).

### Association between inflammatory score and severe malnutrition

In unadjusted models (Model a), moderate and severe inflammatory scores were associated with 24.6% (OR = 1.246, 95% CI: 1.092–1.420, *p* = 0.001) and 1.74-fold (OR = 2.742, 95% CI: 2.422–3.106, *p* < 0.001) increased risks of severe malnutrition (PG-SGA > 8), respectively. Adjustments for age, sex, and TNM stage (Model b) attenuated these risks to 21.2% (OR = 1.212, 95% CI: 1.055–1.393, *p* = 0.007) and 1.491-fold (OR = 2.491, 95% CI: 2.181–2.845, *p* < 0.001). Further adjustment for treatment and comorbidities (Model c) yielded ORs of 1.234 (95% CI: 1.072–1.421, *p* = 0.003) for moderate and 2.553 (95% CI: 2.226–2.927, *p* < 0.001) for severe inflammation (Table 2), confirming the score’s independence from confounders.

**Table 2:** Association between inflammatory score and severe malnutrition (PGSGA > 8) of patients with cancer.

Inflammatory score	Model a [OR, 95% CI]	*p* value	Model b [OR, 95% CI]	*p* value	Model c [OR, 95% CI]	*p* value
Mild	ref	<0.001	ref	<0.001	ref	<0.001
Moderate	1.246 (1.092,1.420)	0.001	1.212 (1.055,1.393)	0.007	1.234 (1.072,1.421)	0.003
Severe	2.742 (2.422,3.106)	<0.001	2.491 (2.181,2.845)	<0.001	2.553 (2.226,2.927)	<0.001

Model a, No adjusted; Model b, Adjusted for age, sex, BMI, TNM stage, tumor types; Model c, Adjusted for age, sex, BMI, TNM stage, tumor types, surgery, radiotherapy, chemotherapy, hypertension, diabetes, smoking, drinking, family history.

### Association between inflammatory score and cachexia

Logistic regression analyses demonstrated strong associations between inflammatory scores and AWGC-defined cachexia. In unadjusted models (Model a), moderate and severe inflammation conferred 54.3% (OR = 1.543, 95% CI: 1.360–1.751, *p* < 0.001) and 1.991-fold (OR = 2.991, 95% CI: 2.643–3.384, *p* < 0.001) risks, respectively. Adjustments for demographics and TNM stage (Model b) yielded ORs of 1.616 (95% CI: 1.412–1.850) and 2.882 (95% CI: 2.523–3.292). Full adjustment (Model c) maintained significance (moderate: OR = 1.535, 95% CI: 1.339–1.760; severe: OR = 2.662, 95% CI: 2.323–3.049; *p* < 0.001; Table 3), validating the score as an independent predictor of cachexia.

**Table 3:** Association between inflammatory score and AWGC cachexia of patients with cancer.

Inflammatory score	Model a [OR, 95% CI]	*p* value	Model b [OR, 95% CI]	*p* value	Model c [OR, 95% CI]	*p* value
Mild	ref	<0.001	ref	<0.001	ref	<0.001
Moderate	1.543 (1.360,1.751)	<0.001	1.616 (1.412,1.850)	<0.001	1.535 (1.339,1.760)	<0.001
Severe	2.991 (2.643,3.384)	<0.001	2.882 (2.523,3.292)	<0.001	2.662 (2.323,3.049)	<0.001

Model a, No adjusted; Model b, Adjusted for age, sex, BMI, TNM stage, tumor types; Model c, Adjusted for age, sex, BMI, TNM stage, tumor types, surgery, radiotherapy, chemotherapy, hypertension, diabetes, smoking, drinking, family history.

### Internal validation of prognostic utility

**Figure 3:**
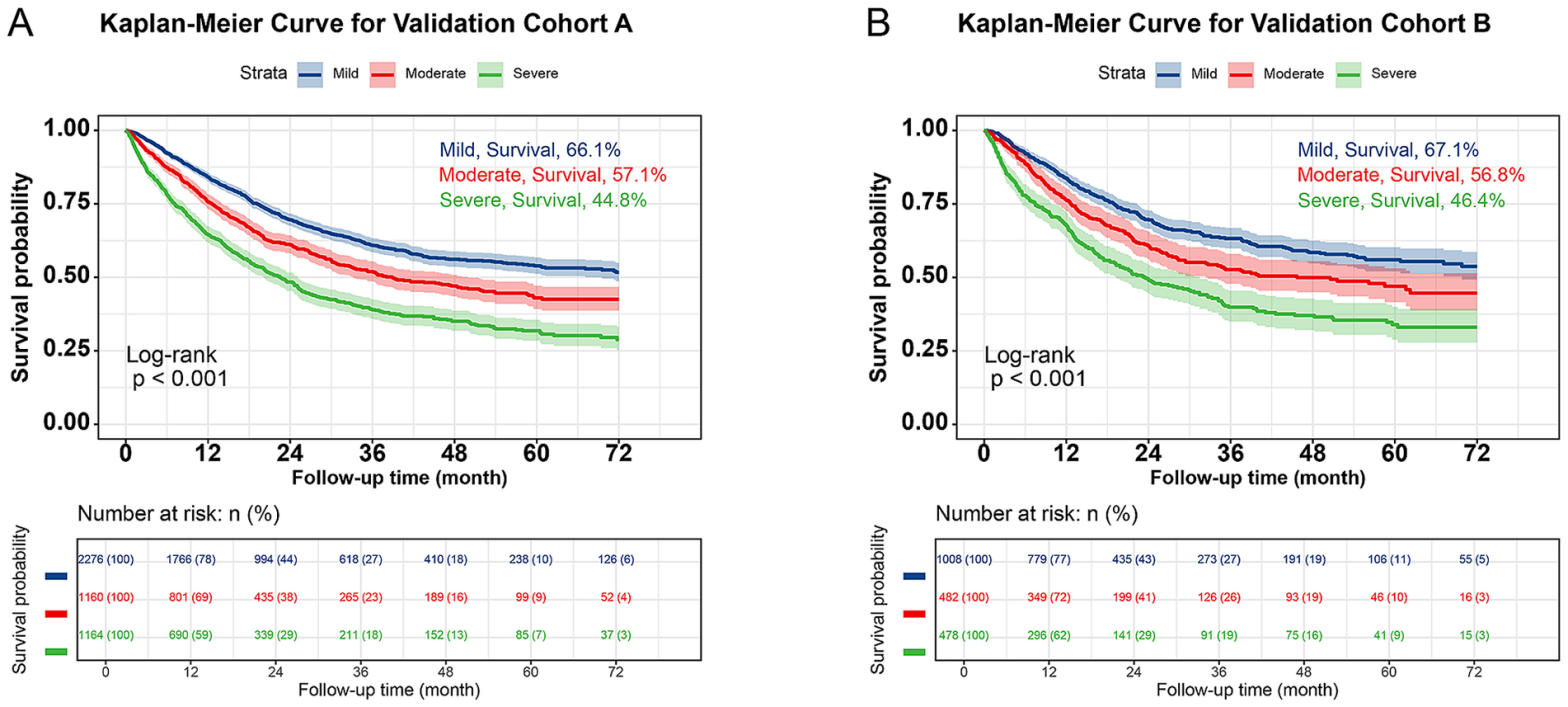
Validation of Kaplan–Meier curve of inflammatory score in patients with cancer. **(A)** Validation cohort A; **(B)** Validation cohort B.

The cohort was randomly split into Validation Cohort A (*n* = 4,600) and B (*n* = 1,968) at the ratio of 7:3, with balanced baseline characteristics (Supplementary Table S5). Two independent validation cohorts demonstrated that the inflammatory score effectively distinguished prognosis in both Validation A (66.1% vs. 57.1% vs. 44.8%, *p* < 0.001) and Validation B (67.1% vs. 56.8% vs. 46.4%, *p* < 0.001). The risk of poor prognosis increased progressively with higher inflammatory scores (Figure 3). In Validation A, fully adjusted Cox proportional hazards analysis revealed that compared with the Mild group, patients in the Moderate group had a 22.5% increased risk of poor prognosis (HR: 1.225, 95% CI: 1.093–1.373, *p* = 0.001), while those in the Severe group had a 61.1% increased risk (HR: 1.611, 95% CI: 1.445–1.797, *p* < 0.001) (Supplementary Table S6). In Validation B, the inflammatory score served as an effective stratification tool for patient prognosis (Moderate: HR: 1.191, 95% CI: 0.998–1.421, *p* = 0.052; Severe: HR: 1.587, 95% CI: 1.341–1.877, *p* < 0.001) (Supplementary Table S7).

## Discussion

The present multicenter cohort study provides compelling evidence that the inflammatory score, derived from baseline WBC and CRP z-scores, is a robust and independent predictor of survival and nutritional deterioration in cancer patients. This finding underscores the critical role of systemic inflammation in cancer progression and highlights the potential clinical utility of the inflammatory score as a prognostic tool and guide for targeted anti-inflammatory therapies.

Chronic inflammation is increasingly recognized as a hallmark of cancer progression, contributing to tumor initiation, growth, and metastasis through complex mechanisms involving immune dysregulation, angiogenesis, and tissue remodeling (4, 21–23). Elevated systemic inflammatory markers, such as WBC and CRP, have been consistently associated with poor prognosis across various malignancies (24–26). The score exhibited a strong dose-dependent relationship with mortality, with severe inflammation conferring a 60.4% increased risk of death compared to mild inflammation in fully adjusted models. Furthermore, subgroup analyses highlighted its enhanced prognostic utility in advanced-stage (III/IV) patients, suggesting potential applications in guiding end-of-life care decisions or eligibility for aggressive therapies. This finding aligns with previous research highlighting the prognostic significance of inflammation in cancer, further emphasizing the need to address systemic inflammation as part of cancer management strategies.

Despite the proliferation of complex molecular prognostic tools in precision oncology, their clinical adoption remains limited by cost, technical complexity, and lack of real-time monitoring capability. In contrast, the inflammatory score leverages two ubiquitously available, low-cost biomarkers (WBC and CRP) that capture systemic inflammation—a universal driver of cancer progression. Unlike tissue-based genomic signatures, this score enables dynamic assessment throughout the treatment trajectory. Crucially, by integrating these non-specific markers via z-score standardization, we overcome the limitations of single-marker variability and provide a holistic quantification of inflammatory burden. Additionally, this study demonstrates that the Inflammatory Score provides superior predictive value for tumor patient survival compared to existing inflammatory markers.

The interplay between chronic inflammation and cancer-related malnutrition is a significant prognostic factor (27, 28). Our study revealed that higher inflammatory scores were strongly associated with severe malnutrition and cachexia, conditions that affect a substantial proportion of cancer patients and are linked to increased mortality. Specifically, severe inflammation was associated with a 1.553-fold increased risk of severe malnutrition and a 1.662-fold increased risk of cachexia. These findings highlight the importance of early identification and management of inflammation to mitigate nutritional deterioration in cancer patients. The inflammatory score may serve as a valuable tool for clinicians to identify patients at high risk of malnutrition and cachexia, facilitating timely interventions to improve outcomes.

The interplay between tumor types and host inflammatory responses exhibits marked heterogeneity, leading to divergent inflammatory burdens across malignancies. In this study, we leveraged inflammatory scores to systematically evaluate the inflammatory landscape of 10 common tumor types, revealing distinct stratification patterns. Notably, we innovatively classified these malignancies into three tiers of inflammatory intensity—low, moderate, and high—based on their systemic inflammatory profiles. This stratification aligns with emerging evidence that tumor-microenvironment crosstalk modulates inflammatory signaling in a cancer-specific manner, driven by variations in cytokine secretion, immune cell infiltration, and metabolic reprogramming (29–32). For instance, gastrointestinal and pancreatic cancers predominantly clustered within the high-inflammatory tier, whereas hormone-sensitive malignancies such as breast and prostate cancers exhibited lower baseline inflammation. Such categorization not only reflects biological diversity but also carries profound clinical implications in the era of precision medicine.

The inflammatory score’s ability to effectively stratify cancer patients based on their inflammatory burden and prognosis suggests its potential as a clinical tool for risk stratification and treatment planning. By identifying patients with severe inflammation, clinicians can prioritize aggressive anti-inflammatory strategies and optimize supportive care to improve survival and quality of life. Furthermore, the score’s association with nutritional outcomes underscores the importance of integrating nutritional support into cancer care pathways, particularly for patients with high inflammatory scores. Future research should focus on validating the inflammatory score in diverse patient populations and exploring its predictive value for specific cancer types and treatment modalities. Additionally, interventional studies are needed to evaluate whether targeting inflammation through pharmacological or nutritional interventions can improve outcomes in patients with high inflammatory scores. The development of standardized protocols for measuring and managing systemic inflammation in cancer care could further enhance the clinical utility of the inflammatory score.

The strengths of this study include its large multicenter cohort, comprehensive data collection, and rigorous statistical analyses. The internal validation using two independent cohorts further supports the robustness of our findings.

However, limitations should be acknowledged. The study’s reliance on observational data precludes definitive causal inferences. Additionally, the inflammatory score’s performance may vary across different ethnicities and cancer types, warranting further validation in diverse populations. Future studies should also consider incorporating additional inflammatory markers and exploring the dynamic changes in the inflammatory score over time to better capture the evolving inflammatory status in cancer patients. While the inability to adjust for all potential confounders represents a limitation of this study, the sequential attenuation of HR from Model a (unadjusted HR = 2.064) to Model b (partially adjusted HR = 1.727) and Model c (fully adjusted HR = 1.604) underscores the presence of significant confounding. Specifically, 33.7% of the excess risk attenuation was attributed to adjustments for TNM stage and tumor types (Model b), with subgroup analyses stratified by these variables confirming the robust prognostic value of the Inflammatory Score. An additional 12.3% attenuation in Model c was driven by treatment modalities (surgery/radiotherapy/chemotherapy) and comorbidities. Although certain critical confounders such as specific chemotherapy regimens were not included, the study has systematically accounted for key prognostic variables within the available dataset, thereby strengthening the validity of the findings. Finally, the borderline significance of moderate inflammation in Validation Cohort B (*p* = 0.052) likely stems from reduced statistical power attributable to its smaller sample size, warranting future validation with a larger cohort.

## Conclusion

The inflammatory score derived from WBC and CRP z-scores is a powerful prognostic indicator in cancer patients, highlighting the critical role of systemic inflammation in cancer progression and nutritional deterioration. Its clinical application holds promise for improving risk stratification and guiding targeted interventions to enhance patient outcomes.

## Data Availability

The original contributions presented in the study are included in the article/Supplementary material, further inquiries can be directed to the corresponding authors.
