# Supplementary material for: Inflammatory score as a predictor of survival and nutritional deterioration in cancer patients: insights from a multicenter cohort study
Source: Front Nutr. 2025 Aug 4;12:1631483. doi: 10.3389/fnut.2025.1631483 (PMC12358745; doi:10.3389/fnut.2025.1631483)
Supplement: Supplementary file 1 [file Data_Sheet_1.docx]

**Figure S1.** Subgroup survival analysis of inflammatory score based on pathological stage.

**
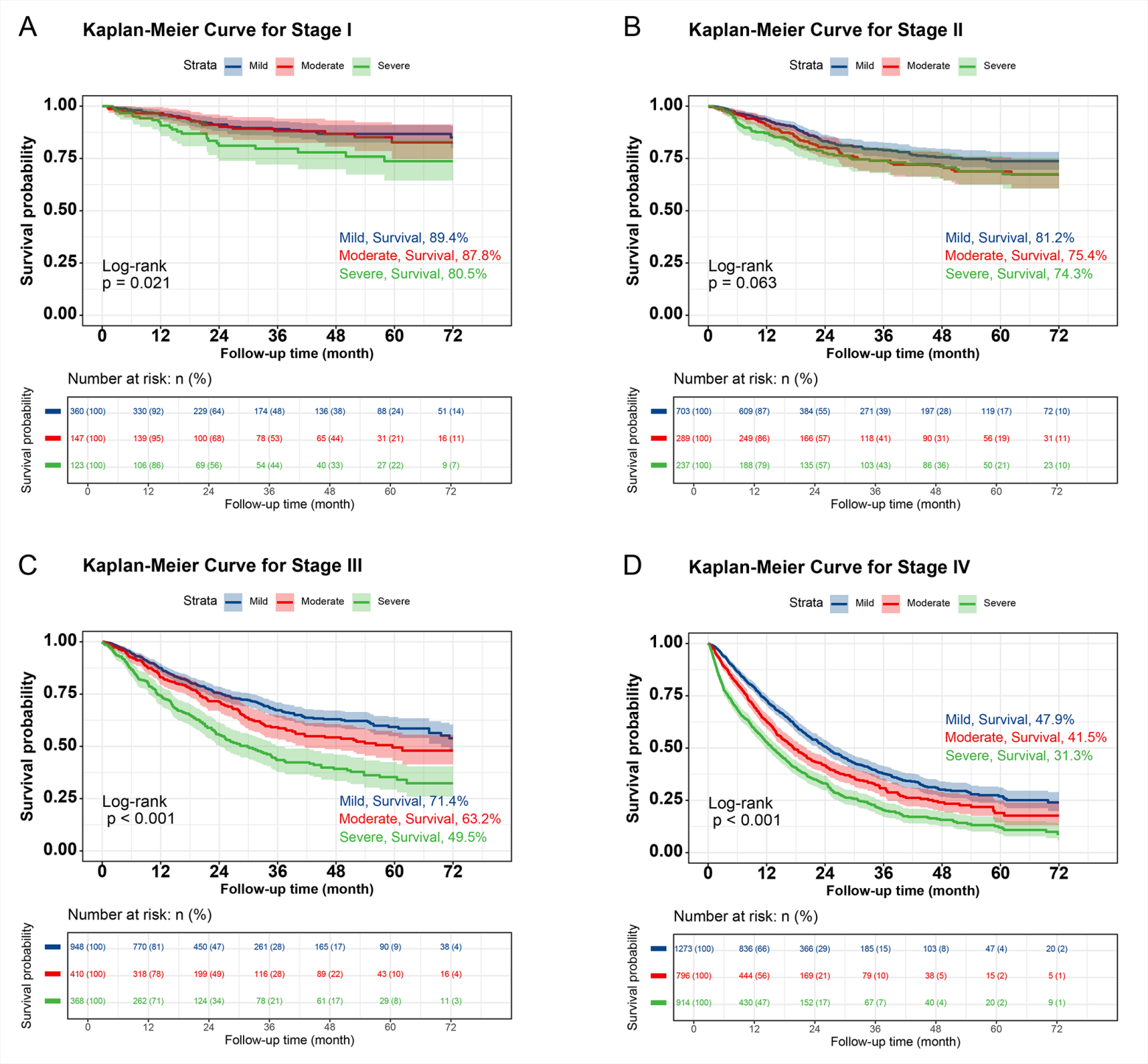
**

**Notes：**A, Stage I; B, Stage II; C, Stage III; D, Stage IV.

**Figure S2.** Subgroup Survival Forest Plot of Different Tumor Types.

**
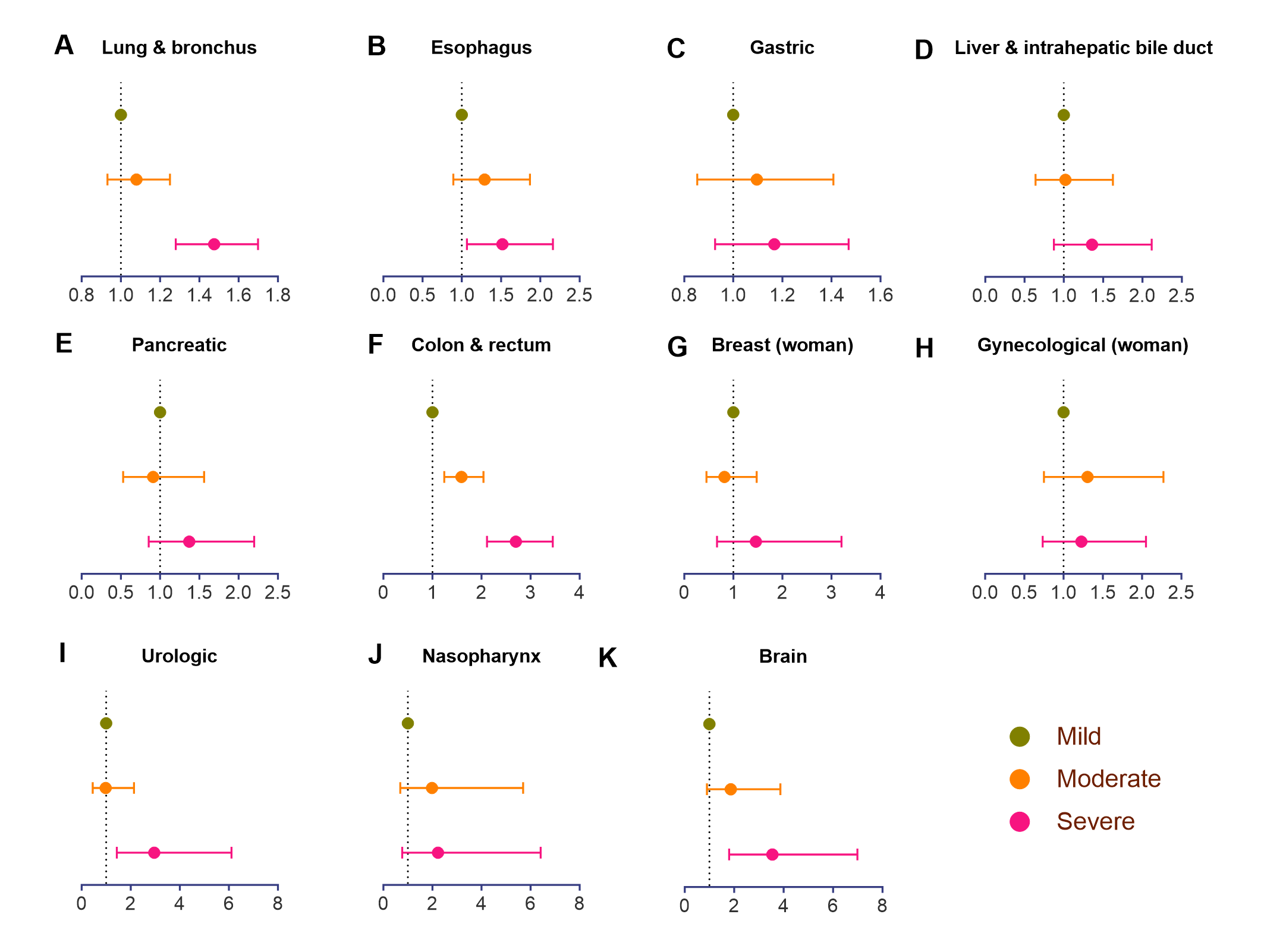
**

**Notes**: A, Lung & bronchus; B, Esophagus; C, Gastric; D, Liver & intrahepatic bile duct; E, Pancreatic; F, Colon & rectum; G, Breast (female); H, Gynecological (female); I, Urologic; J, Nasopharynx; K, Others (brain & other nervous system).

Adjusted for age, sex, BMI, TNM stage, tumor types, surgery, radiotherapy, chemotherapy, hypertension, diabetes, coronary heart disease,smoking, alcohol, family cancer history.

**Figure S3.** Comparison of the prognostic value of composite immune inflammatory indicators by ROC curve.

**
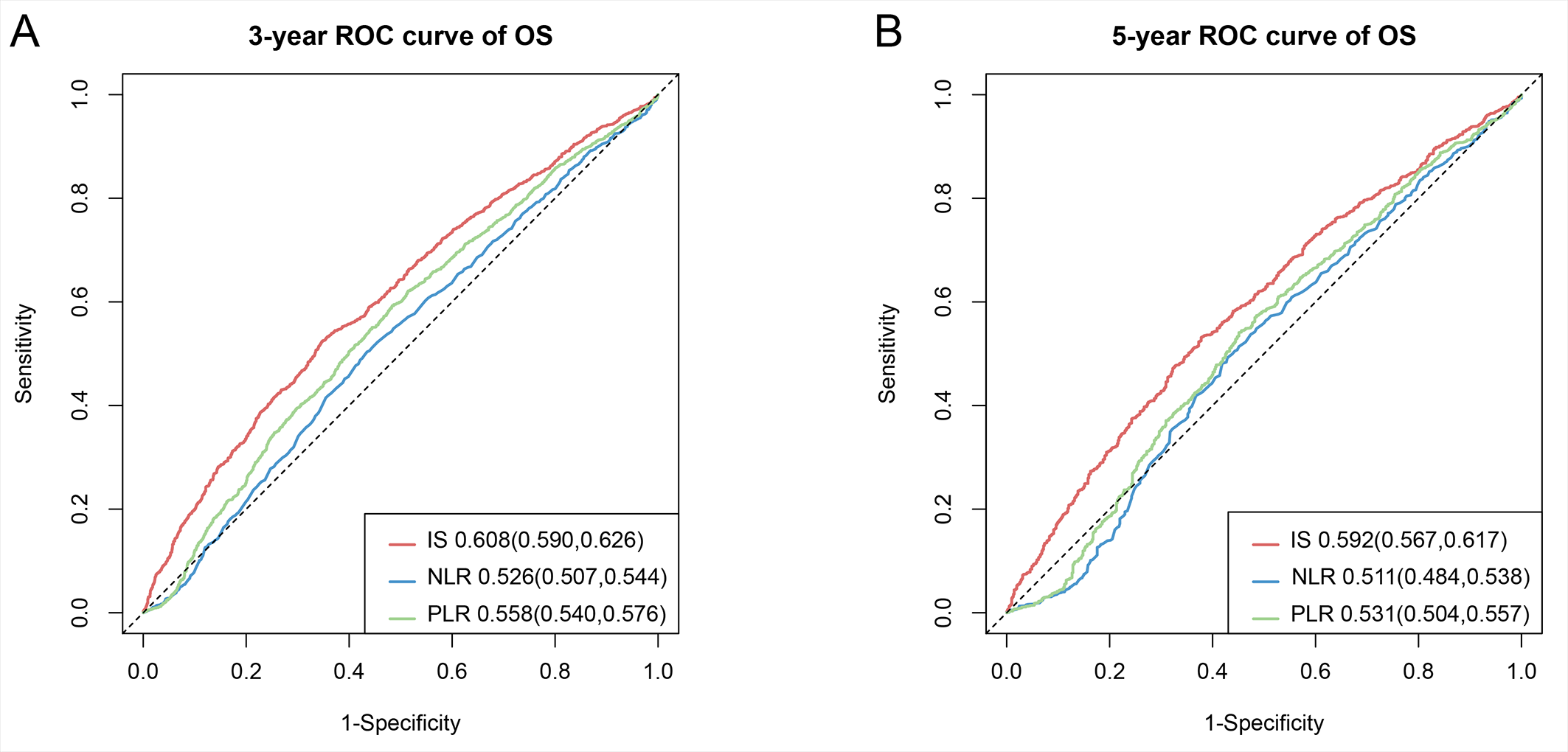
**

**Notes:** A, 3-year ROC curve; B, 5-year ROC curve.

**Figure S4.** The distribution of inflammatory score in different tumor types.

**
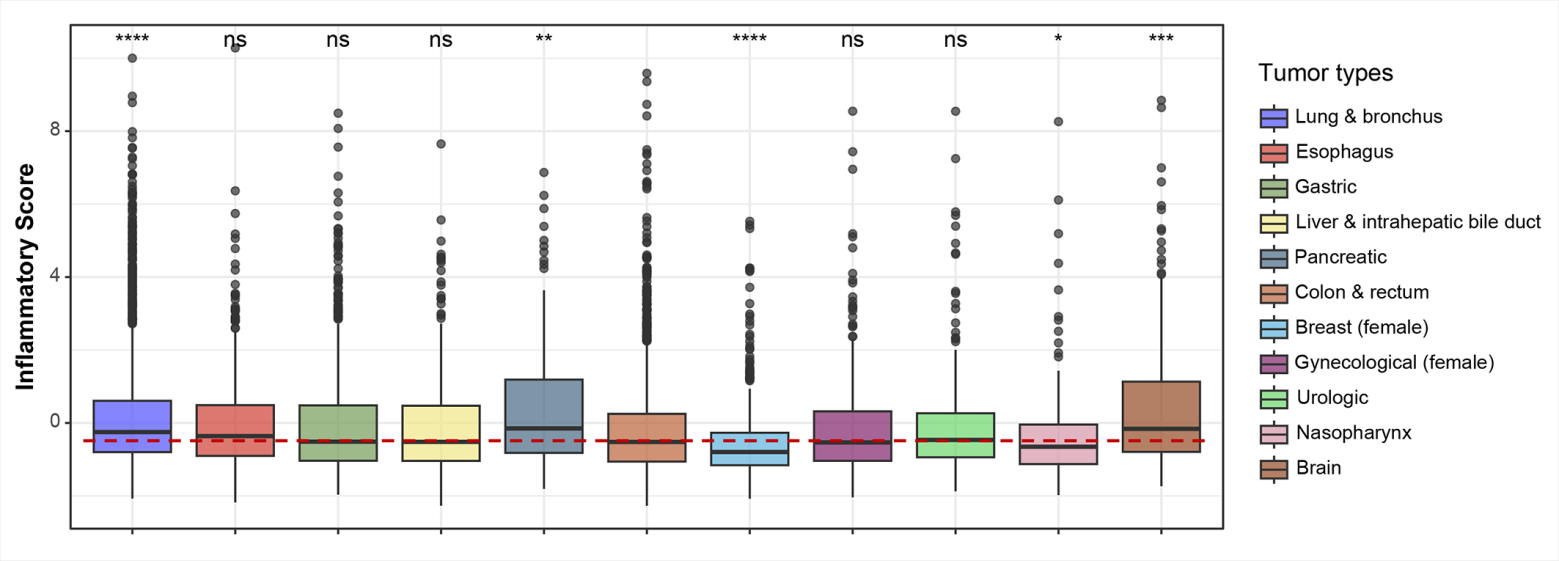
**

**Figure S5.** The distribution of inflammatory score of different cancers at different pathological stages.

**
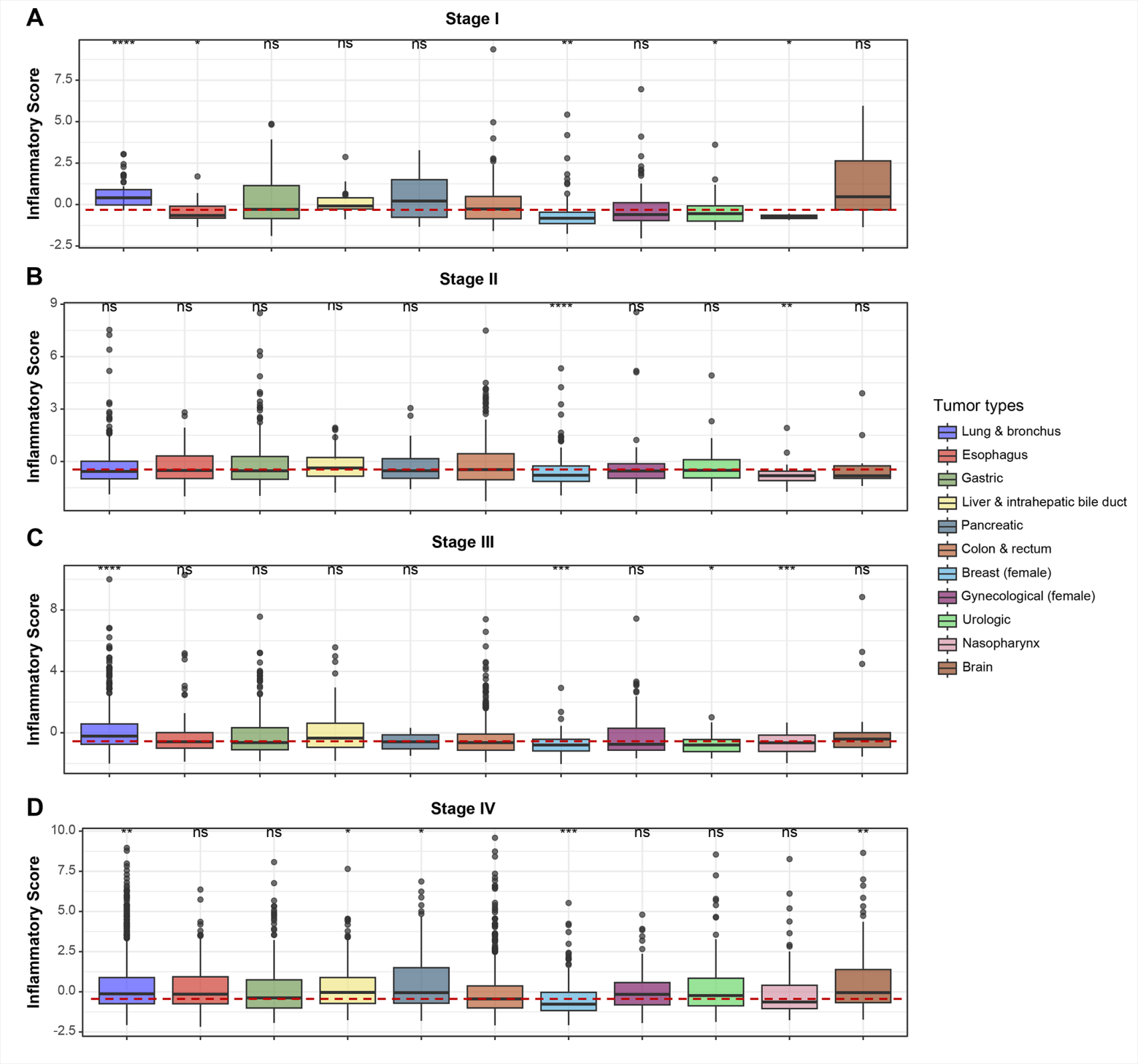
**

**Notes**: A, Stage I; B, Stage II; C, Stage III; D, Stage IV.

**Figure S6.** The tumor inflammation grading system.


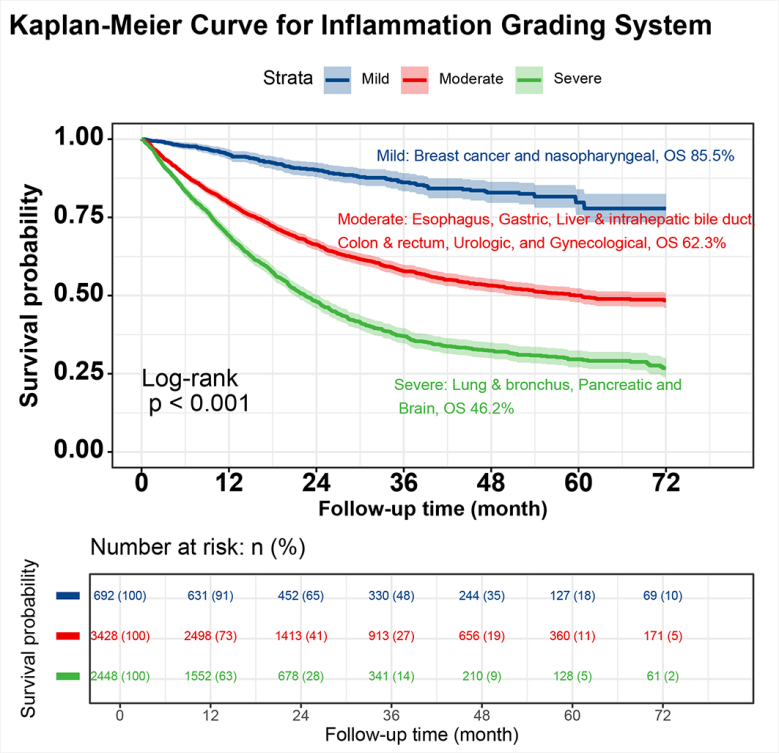


**Notes**: Mild: Breast cancer and nasopharyngeal; B, Moderate: Esophagus, Gastric, Liver & intrahepatic bile duct; Colon & rectum, Urologic, and Gynecological; C, Lung & bronchus, Pancreatic and Brain.

**Table S1.** The names of all participating hospitals.

| Number | Hospitals |
| --- | --- |
| 1 | Anhui Cancer Hospital |
| 2 | Beijing Cancer Hospital |
| 3 | Affiliated Hospital of Chengde Medical College |
| 4 | Tangdu Hospital of Fourth Military Medical University |
| 5 | Foshan First People Hospital |
| 6 | Fujian Cancer Hospital |
| 7 | Guangdong Provincial People Hospital |
| 8 | Guangxi Guigang People Hospital |
| 9 | Guangxi Medical University Affiliated Cancer Hospital |
| 10 | The First Affiliated Hospital of Guangxi Medical University |
| 11 | Guangxi Zhuang People Hospital |
| 12 | Affiliated Cancer Hospital of Zunyi Medical College, Guizhou Province |
| 13 | Affiliated Cancer Hospital of Harbin Medical University |
| 14 | The Fourth Affiliated Hospital of Harbin Medical University |
| 15 | Hebei Provincial People's Hospital |
| 16 | The Second Affiliated Hospital of Hebei Medical University |
| 17 | The Fourth Affiliated Hospital of Hebei Medical University |
| 18 | The First Affiliated Hospital of Hebei Medical University |
| 19 | Huizhou Central People Hospital |
| 20 | Bethune First Hospital of Jilin University |
| 21 | The Third Affiliated Hospital of Kunming Medical University |
| 22 | The First Affiliated Hospital of Kunming Medical University |
| 23 | Liaoning Cancer Hospital |
| 24 | The First Hospital of Shanxi Medical University |
| 25 | Shanghai Ruijin Hospital |
| 26 | Shanghai Tenth People Hospital |
| 27 | Beijing Shijitan Hospital Affiliated to Capital Medical University |
| 28 | West China Hospital of Sichuan University |
| 29 | Sichuan Cancer Hospital |
| 30 | Tianjin Medical University Cancer Hospital |
| 31 | Wuhan Tongji Hospital |
| 32 | Xijing Hospital |
| 33 | The First People Hospital of Kashgar, Xinjiang |
| 34 | Xingtai People's Hospital |
| 35 | Yunnan Cancer Hospital |
| 36 | Yuncheng Central Hospital |
| 37 | The First Affiliated Hospital of Zhejiang University |
| 38 | The Second Affiliated Medical Hospital of Zhejiang University |
| 39 | Zhejiang First Hospital |
| 40 | Zhejiang People's Hospital |
| 41 | Department of Integrated Traditional Chinese and Western Medicine, Zhejiang Cancer Hospital |
| 42 | Cancer Hospital of Chinese Academy of Medical Sciences |

**Table S2.** Characteristics by different levels of Inflammatory score in patients with cancer.

| Characteristic | Overall  (n=6568) | Scores | | |  |
| --- | --- | --- | --- | --- | --- |
|  |  | Mild (n=3284) | Moderate (n=1642) | Severe (n=1642) | p |
| Sex, male, n (%) | 3976 (60.5) | 1811 (55.1) | 1055 (64.3) | 1110 (67.6) | <0.001 |
| Age, years, mean (SD) | 59.45 (11.22) | 58.66 (11.08) | 60.18 (11.25) | 60.31 (11.36) | <0.001 |
| Hypertension, yes, n (%) | 1336 (20.3) | 608 (18.5) | 359 (21.9) | 369 (22.5) | 0.001 |
| Diabetes, yes, n (%) | 678 (10.3) | 305 ( 9.3) | 178 (10.8) | 195 (11.9) | 0.014 |
| Smoking yes, n (%) | 3107 (47.3) | 1357 (41.3) | 834 (50.8) | 916 (55.8) | <0.001 |
| Drinking, yes, n (%) | 1507 (22.9) | 690 (21.0) | 383 (23.3) | 434 (26.4) | <0.001 |
| Family history, yes, n (%) | 1082 (16.5) | 556 (16.9) | 271 (16.5) | 255 (15.5) | 0.458 |
| TNM stage |  |  |  |  | <0.001 |
| Stage I | 630 ( 9.6) | 360 (11.0) | 147 ( 9.0) | 123 ( 7.5) |  |
| Stage II | 1229 (18.7) | 703 (21.4) | 289 (17.6) | 237 (14.4) |  |
| Stage III | 1726 (26.3) | 948 (28.9) | 410 (25.0) | 368 (22.4) |  |
| Stage IV | 2983 (45.4) | 1273 (38.8) | 796 (48.5) | 914 (55.7) |  |
| Tumor.type |  |  |  |  | <0.001 |
| Lung & bronchus | 2115 (32.2) | 894 (27.2) | 604 (36.8) | 617 (37.6) |  |
| Esophagus | 389 ( 5.9) | 185 ( 5.6) | 100 ( 6.1) | 104 ( 6.3) |  |
| Gastric | 1001 (15.2) | 523 (15.9) | 214 (13.0) | 264 (16.1) |  |
| Liver & intrahepatic bile duct | 281 ( 4.3) | 146 ( 4.4) | 63 ( 3.8) | 72 ( 4.4) |  |
| Pancreatic | 153 ( 2.3) | 64 ( 1.9) | 32 ( 1.9) | 57 ( 3.5) |  |
| Colon & rectum | 1291 (19.7) | 690 (21.0) | 318 (19.4) | 283 (17.2) |  |
| Breast (female) | 515 ( 7.8) | 355 (10.8) | 108 ( 6.6) | 52 ( 3.2) |  |
| Gynecological (female) | 257 ( 3.9) | 141 ( 4.3) | 55 ( 3.3) | 61 ( 3.7) |  |
| Urologic | 209 ( 3.2) | 110 ( 3.3) | 53 ( 3.2) | 46 ( 2.8) |  |
| Nasopharynx | 177 ( 2.7) | 109 ( 3.3) | 42 ( 2.6) | 26 ( 1.6) |  |
| Brain | 180 ( 2.7) | 67 ( 2.0) | 53 ( 3.2) | 60 ( 3.7) |  |
| Surgery, yes, n (%) | 3975 (60.5) | 2226 (67.8) | 930 (56.6) | 819 (49.9) | <0.001 |
| Radiotherapy, yes, n (%) | 909 (13.8) | 538 (16.4) | 198 (12.1) | 173 (10.5) | <0.001 |
| Chemotherapy, yes, n (%) | 4438 (67.6) | 2411 (73.4) | 1062 (64.7) | 965 (58.8) | <0.001 |
| White blood cells (median (IQR)) | 6.00 (4.70, 7.80) | 4.80 (3.95, 5.56) | 7.06 (6.50, 7.70) | 9.57 (7.98, 11.50) | <0.001 |
| Neutrophil (median (IQR)) | 3.77 (2.61, 5.40) | 2.77 (2.10, 3.43) | 4.60 (3.90, 5.35) | 6.97 (5.40, 9.09) | <0.001 |
| Lymphocyte (median (IQR)) | 1.43 (1.03, 1.87) | 1.38 (1.03, 1.73) | 1.60 (1.15, 2.09) | 1.43 (0.96, 1.99) | <0.001 |
| Platelets (median (IQR)) | 219.00 (169.00, 281.00) | 199.00 (157.00, 250.00) | 235.00 (186.00, 291.00) | 256.00 (192.00, 336.00) | <0.001 |
| Red blood cells (median (IQR)) | 126.00 (110.00, 138.00) | 126.00 (112.00, 137.00) | 130.00 (114.00, 143.00) | 120.00 (103.00, 136.00) | <0.001 |
| Hemoglobin (median (IQR)) | 4.23 (3.78, 4.63) | 4.20 (3.80, 4.57) | 4.38 (3.93, 4.78) | 4.12 (3.60, 4.59) | <0.001 |
| Albumin (median (IQR)) | 39.40 (35.60, 42.40) | 40.30 (37.20, 43.10) | 39.80 (36.30, 42.60) | 36.10 (32.30, 39.80) | <0.001 |
| PGSGA score (median (IQR)) | 5.00 (2.00, 9.00) | 4.00 (2.00, 8.00) | 5.00 (2.00, 8.00) | 7.00 (3.00, 11.00) | <0.001 |
| NRS2002 | 1.00 (1.00, 3.00) | 1.00 (1.00, 3.00) | 1.00 (1.00, 3.00) | 2.00 (1.00, 4.00) | <0.001 |
| KPS | 90.00 (80.00, 90.00) | 90.00 (80.00, 90.00) | 90.00 (80.00, 90.00) | 80.00 (70.00, 90.00) | <0.001 |
| Cachexia, yes, n (%) | 2362 (36.0) | 894 (27.2) | 601 (36.6) | 867 (52.8) | <0.001 |
| 90-day outcomes | 419 ( 6.4) | 101 ( 3.1) | 104 ( 6.3) | 214 (13.0) | <0.001 |
| Status, death, n (%) | 2709 (41.2) | 1104 (33.6) | 706 (43.0) | 899 (54.8) | <0.001 |
| Length of hospitalization (median (IQR)) | 10.00 (6.00, 16.00) | 9.00 (5.00, 15.00) | 10.00 (7.00, 16.00) | 12.00 (8.00, 18.00) | <0.001 |
| Hospitalization expenses(median (IQR)) | 17161.05 (9877.10, 35542.97) | 15305.70 (8886.75, 29612.00) | 17454.50 (10111.67, 34375.82) | 21698.00 (12194.00, 47703.30) | <0.001 |

**Table S3.** The AIC/BIC of Restricted Cubic Splines.

| Model fit statistics | Model a | | Model b | | Model c | |
| --- | --- | --- | --- | --- | --- | --- |
|  | AIC | BIC | AIC | BIC | AIC | BIC |
| 3 knots | 44601.76 | 44615.34 | 43338.47 | 43379.21 | 43199.29 | 43294.35 |
| 4 knots | 44585.27 | 44605.64 | 43328.94 | 43376.47 | 43190.98 | 43292.83 |
| 5 knots | 44585.82 | 44612.98 | 43330.55 | 43384.87 | 43192.29 | 43300.92 |

**Notes:** AIC, Akaike Information Criterion; BIC, Bayesian Information Criterion.

**Table S4.** Sensitivity analysis was performed by excluding patients with short-term death (419 cases).

| Inflammatory score | Model a [HR, 95% CI] | p value | Model b [HR, 95% CI] | p value | Model c [HR, 95% CI] | p value |
| --- | --- | --- | --- | --- | --- | --- |
| Mild | ref | <0.001 | ref | <0.001 | ref | <0.001 |
| Moderate | 1.321 (1.194,1.461) | <0.001 | 1.218 (1.099,1.35) | <0.001 | 1.151 (1.037,1.277) | 0.008 |
| Severe | 1.788 (1.622,1.971) | <0.001 | 1.505 (1.363,1.662) | <0.001 | 1.389 (1.254,1.538) | <0.001 |

Notes:

Model a: No adjusted.

Model b: Adjusted for age, sex, BMI, TNM stage, tumor types.

Model c: Adjusted for age, sex, BMI, TNM stage, tumor types, surgery, radiotherapy, chemotherapy, hypertension, diabetes, smoking, drinking, family history.

**Table S5.** Characteristics of Validation cohort A and Validation cohort B.

| Characteristic | Validation cohort A (n=4600) | Validation cohort B (n=1968) | p |
| --- | --- | --- | --- |
| Sex, male, n (%) | 2756 (59.9) | 1220 (62.0) | 0.121 |
| Age, years, mean (SD) | 59.34 (11.23) | 59.72 (11.18) | 0.207 |
| Hypertension, yes, n (%) | 924 (20.1) | 412 (20.9) | 0.454 |
| Diabetes, yes, n (%) | 468 (10.2) | 210 (10.7) | 0.574 |
| Smoking yes, n (%) | 2166 (47.1) | 941 (47.8) | 0.607 |
| Drinking, yes, n (%) | 1055 (22.9) | 452 (23.0) | 0.998 |
| Family history, yes, n (%) | 726 (15.8) | 356 (18.1) | 0.023 |
| TNM stage |  |  | 0.362 |
| Stage I | 439 ( 9.5) | 191 ( 9.7) |  |
| Stage II | 844 (18.3) | 385 (19.6) |  |
| Stage III | 1196 (26.0) | 530 (26.9) |  |
| Stage IV | 2121 (46.1) | 862 (43.8) |  |
| Tumor.type |  |  | 0.361 |
| Lung & bronchus | 1469 (31.9) | 646 (32.8) |  |
| Esophagus | 263 ( 5.7) | 126 ( 6.4) |  |
| Gastric | 724 (15.7) | 277 (14.1) |  |
| Liver & intrahepatic bile duct | 197 ( 4.3) | 84 ( 4.3) |  |
| Pancreatic | 112 ( 2.4) | 41 ( 2.1) |  |
| Colon & rectum | 893 (19.4) | 398 (20.2) |  |
| Breast (female) | 357 ( 7.8) | 158 ( 8.0) |  |
| Gynecological (female) | 183 ( 4.0) | 74 ( 3.8) |  |
| Urologic | 140 ( 3.0) | 69 ( 3.5) |  |
| Nasopharynx | 137 ( 3.0) | 40 ( 2.0) |  |
| Brain | 125 ( 2.7) | 55 ( 2.8) |  |
| Surgery, yes, n (%) | 2769 (60.2) | 1206 (61.3) | 0.426 |
| Radiotherapy, yes, n (%) | 622 (13.5) | 287 (14.6) | 0.27 |
| Chemotherapy, yes, n (%) | 3108 (67.6) | 1330 (67.6) | 1 |
| White blood cells (median (IQR)) | 6.00 (4.70, 7.75) | 6.00 (4.68, 7.90) | 0.92 |
| Neutrophil (median (IQR)) | 3.77 (2.63, 5.38) | 3.74 (2.60, 5.46) | 0.837 |
| Lymphocyte (median (IQR)) | 1.43 (1.04, 1.87) | 1.46 (1.03, 1.89) | 0.386 |
| Platelets (median (IQR)) | 220.00 (170.00, 280.25) | 218.00 (168.00, 281.00) | 0.554 |
| Red blood cells (median (IQR)) | 126.00 (110.00, 138.00) | 125.00 (111.00, 138.00) | 0.71 |
| Hemoglobin (median (IQR)) | 4.22 (3.78, 4.63) | 4.24 (3.80, 4.64) | 0.54 |
| Albumin (median (IQR)) | 39.30 (35.50, 42.30) | 39.40 (35.70, 42.50) | 0.522 |
| PGSGA score (median (IQR)) | 5.00 (2.00, 9.00) | 5.00 (2.00, 9.00) | 0.351 |
| NRS2002 | 1.00 (1.00, 3.00) | 1.00 (1.00, 4.00) | 0.168 |
| KPS | 90.00 (80.00, 90.00) | 90.00 (80.00, 90.00) | 0.21 |
| Cachexia, yes, n (%) | 1655 (36.0) | 707 (35.9) | 0.989 |
| 90-day outcomes | 305 ( 6.6) | 114 ( 5.8) | 0.223 |
| Status, death, n (%) | 1913 (41.6) | 796 (40.4) | 0.405 |
| Length of hospitalization (median (IQR)) | 10.00 (6.00, 16.00) | 10.00 (6.00, 16.00) | 0.219 |
| Hospitalization expenses(median (IQR)) | 17002.00 (9845.28, 34954.53) | 17472.55 (9982.65, 36922.45) | 0.136 |

**Table S6.** Inflammatory score at Validation cohort A.

| Inflammatory score | Model a [HR, 95% CI] | p value | Model b [HR, 95% CI] | p value | Model c [HR, 95% CI] | p value |
| --- | --- | --- | --- | --- | --- | --- |
| Mild | ref | <0.001 | ref | <0.001 | ref | <0.001 |
| Moderate | 1.399 (1.25,1.566) | <0.001 | 1.278 (1.141,1.431) | <0.001 | 1.225 (1.093,1.373) | 0.001 |
| Severe | 2.082 (1.875,2.312) | <0.001 | 1.732 (1.557,1.925) | <0.001 | 1.611 (1.445,1.797) | <0.001 |

Notes:

Model a: No adjusted.

Model b: Adjusted for age, sex, BMI, TNM stage, tumor types.

Model c: Adjusted for age, sex, BMI, TNM stage, tumor types, surgery, radiotherapy, chemotherapy, hypertension, diabetes, smoking, drinking, family history.

**Table S7.** Inflammatory score at Validation cohort B.

| Inflammatory score | Model a [HR, 95% CI] | p value | Model b [HR, 95% CI] | p value | Model c [HR, 95% CI] | p value |
| --- | --- | --- | --- | --- | --- | --- |
| Mild | ref | <0.001 | ref | <0.001 | ref | <0.001 |
| Moderate | 1.372 (1.154,1.632) | <0.001 | 1.274 (1.07,1.517) | 0.006 | 1.191 (0.998,1.421) | 0.052 |
| Severe | 2.02 (1.716,2.378) | <0.001 | 1.716 (1.455,2.023) | <0.001 | 1.587 (1.341,1.877) | <0.001 |

Notes:

Model a: No adjusted.

Model b: Adjusted for age, sex, BMI, TNM stage, tumor types.

Model c: Adjusted for age, sex, BMI, TNM stage, tumor types, surgery, radiotherapy, chemotherapy, hypertension, diabetes, smoking, drinking, family history.
